# MUC4 is not expressed in cell lines used for live cell imaging

**DOI:** 10.12688/wellcomeopenres.17229.2

**Published:** 2021-11-19

**Authors:** Naouel Athmane, Iain Williamson, Shelagh Boyle, Simon C. Biddie, Wendy A. Bickmore

**Affiliations:** 1 MRC Human Genetics Unit, Institute of Genetics and Cancer, University of Edinburgh, Edinburgh, Scotland, EH42XU, UK

**Keywords:** dCas9, imaging, mucin, transcription

## Abstract

**Background: **The ability to visualise specific mammalian gene loci in living cells is important for understanding the dynamic processes linked to transcription. However, some of the tools used to target mammalian genes for live cell imaging, such as dCas9, have been reported to themselves impede processes linked to transcription. The *MUC4* gene is a popular target for live cell imaging studies due to the repetitive nature of sequences within some exons of this gene.

**Methods:** We set out to compare the impact of dCas9 and TALE-based imaging tools on *MUC4* expression, including in human cell lines previously reported as expressing *MUC4*.

**Results**: We were unable to detect *MUC4* mRNA in these cell lines. Moreover, analysis of publicly available data for histone modifications associated with transcription, and data for transcription itself, indicate that neither *MUC4*, nor any of the mucin gene family are significantly expressed in the cell lines where dCas9 targeting has been reported to repress *MUC4* and *MUC1* expression, or in the cell lines where dCas13 has been used to report *MUC4* RNA detection in live cells.

**Conclusions: **Methods for visualising specific gene loci and gene transcripts in live human cells are very challenging. Our data suggest that care should be given to the choice of the most appropriate cell lines for these analyses and that orthogonal methods of assaying gene expression be carefully compared.

## Introduction

Live cell imaging of actively transcribing genes allows investigation of the changes in chromatin dynamics associated with gene expression. However, achieving this involves multiple technical challenges and has, to date, resulted in contradictory conclusions likely due to the different approaches used for visualising the loci under investigation.

Live imaging studies of genomic loci in mammalian cells have mainly targeted repetitive elements. Initial studies relied on the insertion of repetitive arrays of bacterial operators (LacO, tetO, CuO) into the mouse or human genome and visualisation using the corresponding binding proteins (lacI, TetR, CymR) ( Alexander *et al.*, 2019; Chubb *et al.*, 2002; Pollex & Heard, 2019). Repeats are easier to visualise because they help to accumulate many fluorescent molecules in one spot, producing a high signal to noise ratio. The orthogonal ANCHOR system uses insertion of a short ANCH sequence and then spreading of the bacterial ParB protein, seeded at these binding sites, to enhance the signal ( Germier *et al.*, 2017). However, these approaches raise concerns about alteration of the chromatin and epigenetic state of the tagged loci caused by the insertion of bacterial DNA sequences. ( Jacome & Fernandez-Capetillo, 2011; Osorio-Valeriano *et al.*, 2019). 

More recently, directed binding by catalytically dead Cas9 (dCas9) and transcription-activator like effectors (TALEs) have been used to visualise telomeres, centromeres, sub-telomeric and pericentromeric repeats ( Ma *et al.*, 2016) or SINE elements in mammalian cells ( Chen *et al.*, 2013; Knight *et al.*, 2015; Miyanari *et al.*, 2013). These approaches do not rely on modifying the genomic locus under investigation.

However, the goal has been to image specific gene loci – not just repetitive elements. Various approaches, such as the use of multiple sgRNAs and fluorescently-tagged dCas9 ( Gu *et al.*, 2018), or the recruitment of multiple tagged proteins to sgRNAs ( Cheng *et al.*, 2016; Qin *et al.*, 2017) have been used to overcome the signal:noise problem. In addition, the *MUC4* gene has become a popular locus targeted for live cell visualisation due to a repetitive region in one of its exons ( Chen *et al.*, 2013).

However, dCas9 has been reported to inhibit both gene expression when bound at a gene locus ( Chen *et al.*, 2013), and the binding of endogenous transcription factors ( Gao *et al.*, 2014) ( Shariati *et al.*, 2019). Understanding how current live cell imaging tools impact gene expression is therefore an important aim. We set out to compare dCas9 and TALE based imaging to visualise the *MUC4* locus in human cell lines in order to assess the impact of dCas9 and TALE binding on the ability of the targeted locus to be transcribed. However, this work led us to question whether the cell lines commonly used for imaging *MUC4* are an appropriate background on which to investigate *MUC4* expression.

Our experimental data and our bioinformatic analysis of publicly available data suggest that neither *MUC4*, *MUC1* or indeed any member of the mucin gene family, are expressed at significant levels in the cell lines that have been previously used either to study dCas9-mediated repression of *MUC4*/ *MUC1* expression, or to visualise *MUC4* RNA in the nucleus.

## Methods

### Cell culture and transfections

U2OS cells were grown in McCoy’s 5A supplemented with 10% fetal bovine serum (FBS) and Penicillin/Streptomycin. Transfections were performed using Lipofectamine 3000 reagent (Invitrogen) following the manufacturer’s recommendations. Briefly, 90% confluent U2OS were transfected in a 6-well plate with 1μg of plasmid and 3,75μl of lipofectamine and 4 μl of P3000 reagent. dCas9-EGFP or MUC4 targeting TALE-EGFP were transiently transfected into U2OS cells prior to imaging and RNA extraction. Transient transfection of sgRNAs in pSLQ1651 was monitored by mCherry expression.

### DNA FISH

FISH on metaphase arrested U2OS cells was performed as previously described ( Fantes *et al.*, 1992). Fosmid WI2-1916J7 (chr3:195764450-195798680; hg38) was used to detect MUC4 exon 2 and was directly labelled with ChromaTide Alexa Fluor 594-5-dUTP (Thermofisher scientific C11400) by nick translation. 200ng of labelled probe were used per slide, with 8ug human CotI DNA (Invitrogen, cat#18440-016) and 10ug sonicated salmon sperm DNA (Sigma, cat#31149) and denatured in hybridization mix at 70°C for five minutes, then preannealed at 37°C for 15 minutes. The probe was then hybridized to the denatured slides in a humid chamber at 37°C overnight (approximately 16 hours). Slides were washed for 4x3 minutes in 2xSSC at 45°C, then 0.1xSSC at 60°C. Slides were counterstained in 0.5 µg/ml DAPI and mounted using Vectashield prior to imaging.

### dCas9 and TALE *MUC4* targeting constructs

A TALE binding domain targeting *MUC4* was assembled simultaneously using Golden Gate Assembly of Esp3I digested fragments. Four modules from RVD encoding plasmids (Addgene kit #1000000024) based on the MUC4 targeting RVDI to IV described by Ren *et al.* (2017) were assembled into a pTAL-spec-puro-eGFPmodified vector by thermocycling ((37°C 10 mins, 16°C 10 mins)x12), 36°C 15mins, 80°C 5 mins. RVDI(CCTG), RVDII (TCAC), RVDIII (CGAC), RVD IV (ACT). Golden Gate products were transformed into *E. coli* and selected on spectinomycin plates. Colonies screened for fully assembled TALEs by PCR were confirmed by Sanger sequencing and by a diagnostic digestion with NotI and BamHI.

The sgRNA targeting *MUC4* expressed under control of the U6 promoter (pSLQ 1661-sgMUC4 – E3(F+E)) was generated by Chen *et al.* (2013) and ordered from Addgene (addgene #51025). This was co-transfected into U2OS cells together with dCas9-EGFP-NLS plasmid.

### RNA extraction and cDNA synthesis

To quantify gene expression when targeting *MUC4*, dCas9-EGFP and TALE-EGFP expressing cells were harvested 24h after gRNA transfection. RNA was extracted from approximately 1×10 ^6^ cell pellets using the RNeasy mini kit (Qiagen 74106) as per the manufacturer’s instruction, including an on-column DNase digestion (Qiagen 79254), eluted in 20 μL ddH2O and quantified using the Qubit RNA broad range assay (ThermoFisher Q10210) with the Qubit 4 fluorometer. cDNA was synthesised from 2 μg RNA using Superscript II reverse transcriptase (Invitrogen 18064071) primed with random hexamers in a final volume of 20µl (Promega C1181) as per the manufacturer’s instructions.

### RT-qPCR

**Figure 1.**
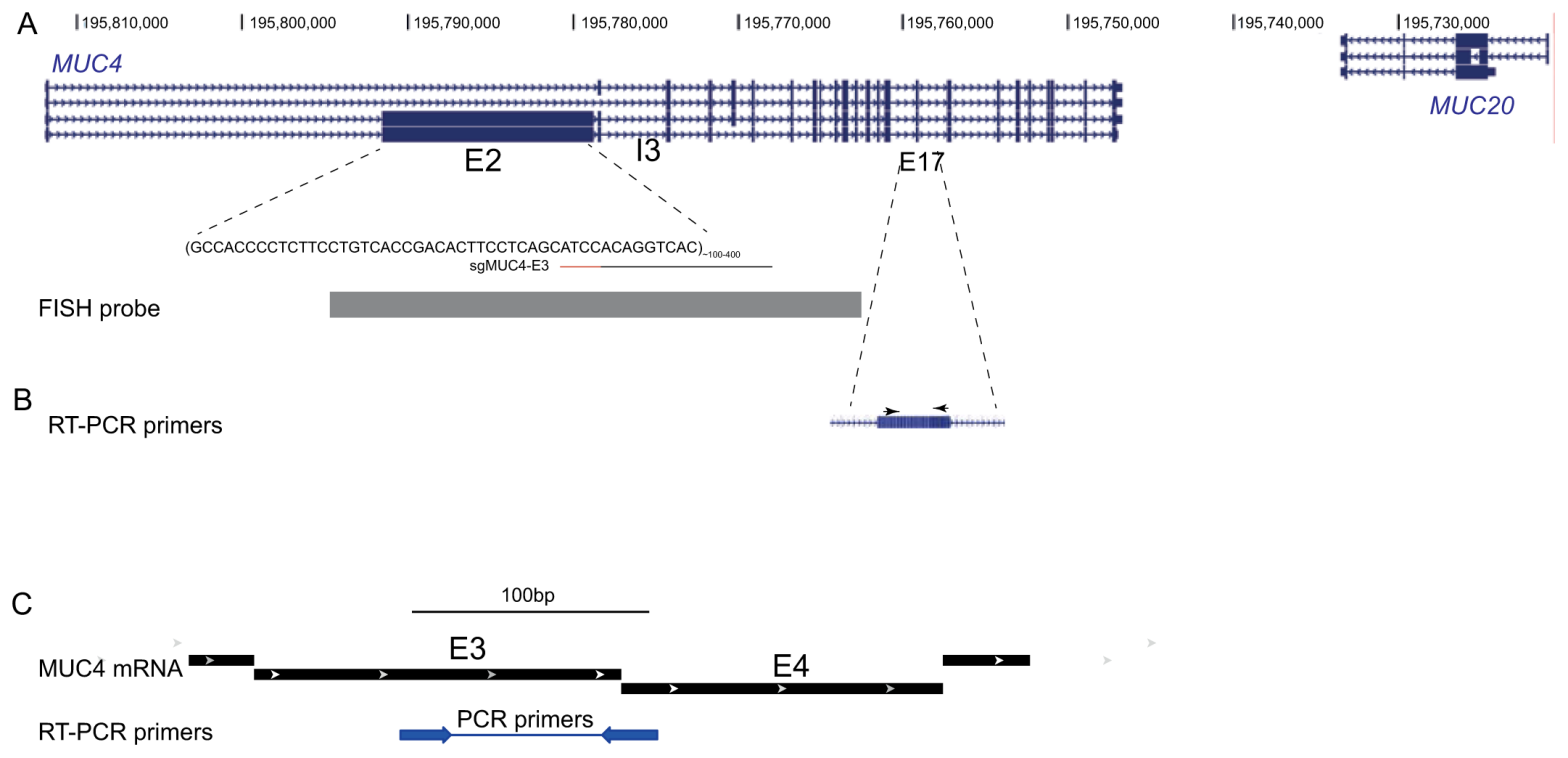
The *MUC4* locus. ( **A**) UCSC Genome Browser screen shot of the MUC4/MUC20 region on human chromosome 3. The location of the two repeated regions in exon2 (E2) and intron3 (I3) of MUC4 are indicated. The target sequence (black line) and the PAM (red line) of sgMUC4-E3 for targeting of dCas9 to E2 of MUC4 are shown. At the bottom, the position of the probe used for FISH is indicated. ( **B**) Location of primers (arrowheads) located within exon 17 used for RT-PCR analysis of MUC4 expression by Chen *et al.* (2013). ( **C**) Location of primers (arrowheads) spanning an exon-exon junction used here for RT-PCR analysis of MUC4 mRNA expression (mRNA transcript variant used for design: NCBI Reference Sequence: NM_004532.6).

For real-time (q)RT-PCR analysis of *MUC4* expression in U2OS cells ( Figure 3A and B), qPCR was carried out on the BioRad CFX96 Real-Time System as follows: For a final volume of 10 µl, three technical replicates were prepared together for each sample: 17.5 µl of Light cycler 480 SYBR green I master (Roche 04887352001) + 10.5µl 1 µM primer mix of forward and reverse primer + 7 µl cDNA (diluted 1:4). A standard curve was included for each primer set. Thermal cycler conditions were 44 PCR cycles (95° for 5 min, 95° for 10s, 60° for 10s, 72° for 20s). Primer sequences used for qRT-PCR were: 

*MUC4* ( Chen *et al.*, 2013) ( Figure 1B)

Fw: 5’ TCAATGGTGGTCGTGTGATT 3’

Rv: 5’ AAGTCGGTGCAGCTGTCTCT 3’

*β-actin*

Fw 5’ CATGTACGTTGCTATCCAGGC 3’

Rv 5’ CTCCTTAATGTCACGCACGAT 3’

Gene expression data were analysed for transfected cells (TALE or dCas9) vs mock transfected cells, normalised to β-actin by the (2 ^– *ΔΔC _T_*^) method ( Livak & Schmittgen, 2001) as follows:

$$\Delta \Delta C_{T}=[(C_{T}MUC4-C_{T}\beta actin)transfected-(C_{T}MUC4-C_{T}\beta actin)mock]$$

### RT-PCR

First strand product was amplified by PCR using primers spanning between exon 3 and exon 4 of *MUC4* for 35 cycles (95°C for 20 s, 60°C for 30 s, 72°C for 30 s) and products visualised by agarose gel electrophoresis ( Figure 3C). A -RT control was included. Primer sequences were:

MUC4 ( Figure 1C):

Fw: 5’ CACAACCTCCCAGACCATCAT 3’

Rv: 5’ GGAAGAGGGAAACTCCTCTCTCA 3’

β-actin:

Fw 5’ AGAGCTATGAGCTGCCTGACG 3’

Rv 5’ TGTGTTGGCATAGAGGTCTTTACG 3’

### Image acquisition

U2OS cells growing on slides were fixed with 4 % paraformaledhyde, permeabilised with Triton X-100 and DAPI stained 24h following transfection with dCas9 or TALE constructs. Slides were imaged using a Photometrics Coolsnap HQ2 CCD camera and a Zeiss AxioImager A1 fluorescence microscope with a Plan Apochromat 100x 1.4NA objective, a Nikon Intensilight Mercury based light source (Nikon UK Ltd, Kingston-on-Thames, UK) and Chroma #89014ET (3 colour) single excitation and emission filters (Chroma Technology Corp., Rockingham, VT) with the excitation and emission filters installed in Prior motorised filter wheels. A piezoelectrically driven objective mount (PIFOC model P-721, Physik Instrumente GmbH & Co, Karlsruhe) was used to control movement in the z dimension. Step size for z stacks was set to 0.2 μm. Hardware control and image capture were performed the acquisition module or Nikon Nis-Elements software (Nikon UK Ltd, Kingston-on-Thames, UK).

### Bioinformatic analysis of gene expression

ENCODE chromatin immunoprecipitation (ChIP)-seq data from U2OS cells were obtained from NCBI GEO: H3K4me3 (GSM871043), H3K36me3 (GSM788076).

For mature and nascent RNA-seq analysis from cell lines, publicly available data were obtained as indicated in Table 1. FastQ files were aligned to human genome hg38 using Bowtie.2 with default settings. BAM files were then used to generate BigWig files using bamCoverage with normalisation across samples by scaling to 1X genome size. 

**Table 1.** Sources of mRNA and nascent RNA-seq data for Capan-1 and 2 cells, U2OS, RPE1, HeLa and HT1080 cells.

Cell line	RNA	GSE Series	SRA Number
Mature mRNA			
Capan-1	mRNA	GSE79669	SRR3308945
Capan-2	mRNA	GSE79669	SRR3308945
U-2OS	mRNA	GSE162163	SRR13142368
RPE1	mRNA	GSE98541	SRR5508027
HeLa	mRNA	GSE90235	SRR5048095
HT1080	mRNA	GSE78653	SRR3192620
**Nascent RNA**			
U-2OS	4SU-seq (4-thiouridine)	GSE162264	SRR13159400
RPE1	EU-seq (5-ethynyl uridine)	GSE137448	SRR10119526
HeLa	4SU-seq (4-thiouridine)	GSE128753	SRR8775198

To analyse mucin gene family expression, transcripts per million (TPM) data from RNA-seq datasets for 934 cell lines were obtained from the EBI-EMBL Expression Atlas data release 37. A heatmap was generated using *pheatmap* with TPM counts expressed as a Z-score. Cell lines were assigned an organ origin type with clustering using the default clustering method.

## Results

### Detection of the *MUC4* locus in U2OS cells

*MUC4* is the most common mammalian gene targeted for visualisation in live cells as its coding sequence has a variable number (>100) of a 48 nt tandem repeat in exon 2. This 7.5 to 19 kb repeat region results in the translation of a 550 to 930 kDa protein ( Chaturvedi *et al.*, 2008). The *MUC4* locus also contains 90 repeats of a 15 bp sequence in intron 3 ( Chen *et al.*, 2013) ( Figure 1).

Because dCas9 and TALE targeting approaches have been shown to have very different efficiencies with regard to the synthetic activation and repression of gene loci ( Gao *et al.*, 2014), we aimed to target dCas9-GFP or TALE-GFP to the *MUC4* locus in human cell lines with the aim of assaying the chromatin dynamics of this locus when transcriptionally active.

There have been various reports visualising *MUC4* in live U2OS cells ( Chen *et al.*, 2013; Qin *et al.*, 2017). We therefore chose this bone osteosarcoma epithelial cell line for our initial studies. We targeted dCas9, to the *MUC4* locus using a sgRNA ( Figure 1A) previously designed for exon 2 ( Chen *et al.*, 2013). We also designed TALE proteins that target exon 2 of MUC4.

**Figure 2.**
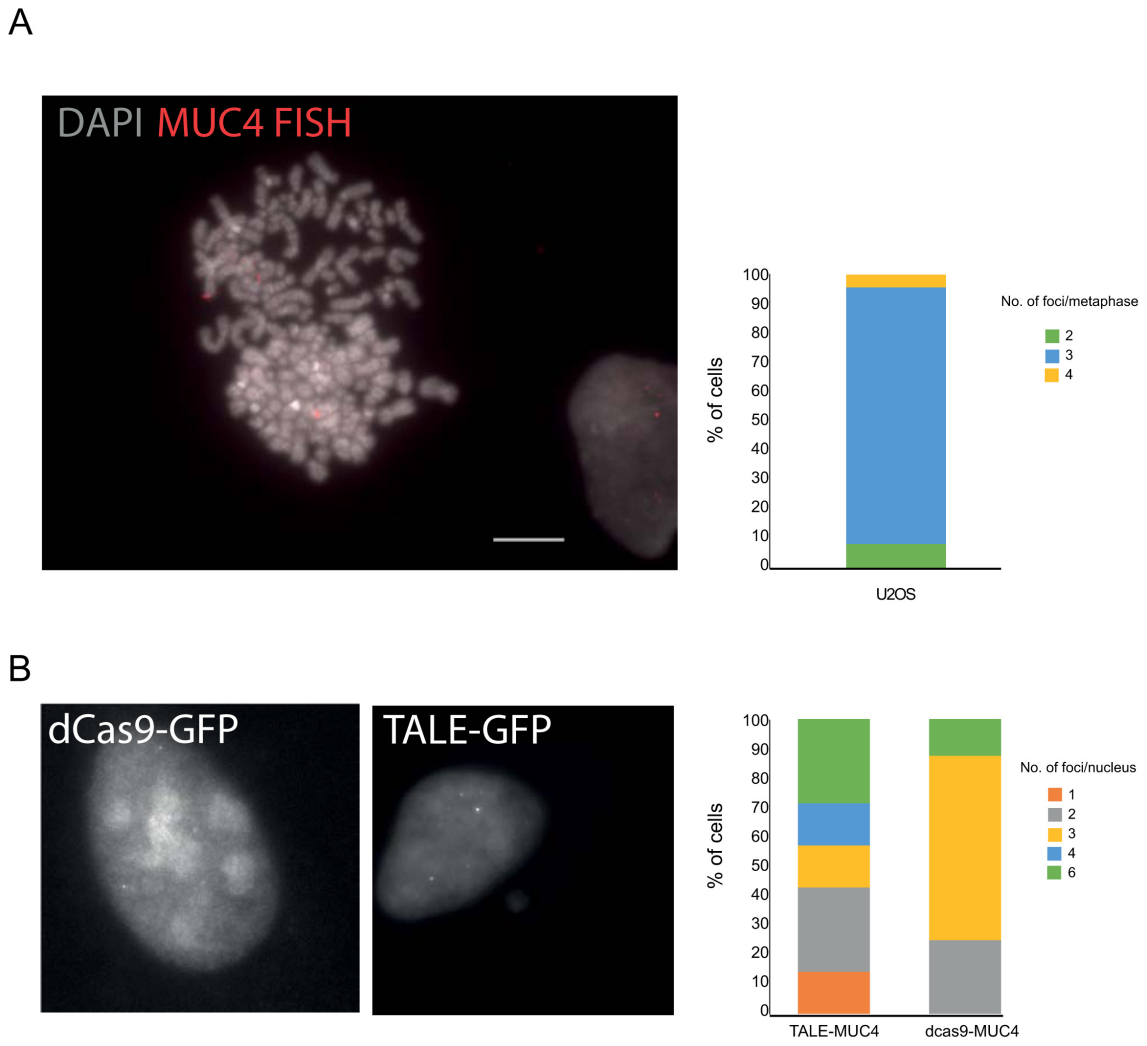
Detection of the *MUC4* locus. **A**) Representative FISH image of a U2OS DAPI-stained metaphase chromosomes hybridised with the MUC4 fosmid probe (red) and indicating three copies of the locus. Scale bar= 10μm. The graph to the right shows the number of loci detected per spread (n=23 metaphase spreads). **B**) Images of GFP fluorescence in fixed U2OS cells transiently expressing either dCas9-gRNA or TALE constructs specific to the *MUC4* locus. The graphs to the right quantify the number of spots observed in transfected cells for either dCas9 or TALE.

U2OS cells are reported to be hypertriploid. We confirmed this for chromosome 3q, where *MUC4* is located, using DNA FISH ( Figure 2A) with a probe that encompasses exon 3 ( Figure 1A). MUC4-specific dCas9+sgRNA as well as TALE-GFP constructs were then transfected into U2OS cells. Foci were detected with both dCas9 and TALES. However, whilst 3 spots per nucleus were detected by the dCas9, consistent with the presence of three copies of the locus detected by DNA FISH ( Figure 2B), up to 6 spots per nucleus were detected with the TALE-GFPs leading us to question what the TALE constructs were detecting.

### *MUC4* is not expressed in U2OS, RPE or HeLa cells

**Figure 3.**
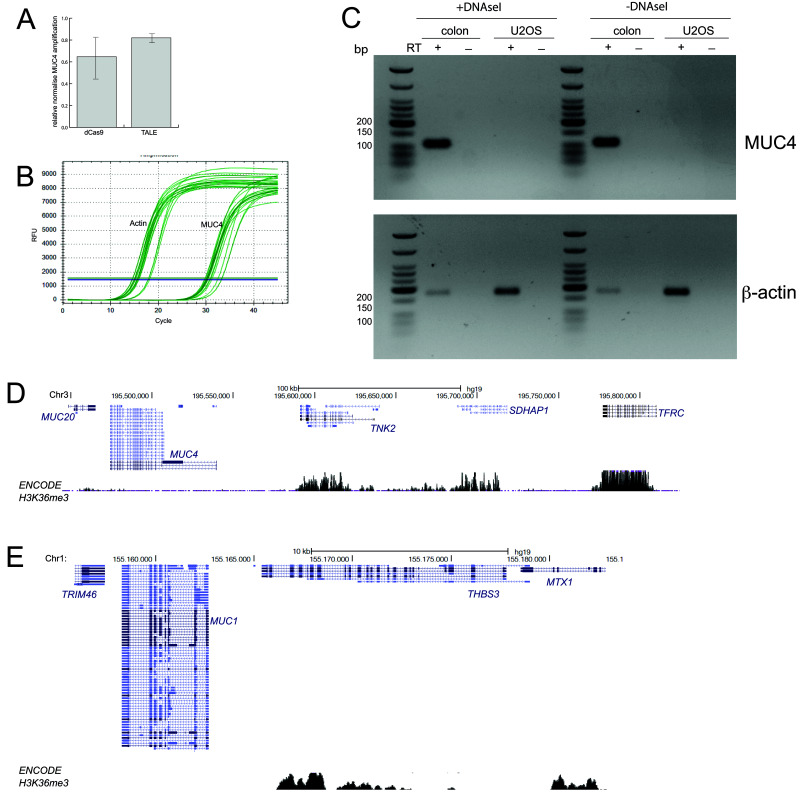
No evidence of *MUC4* expression in U2OS cells. **A**) qRT-PCR data for *MUC4* showing amplification levels in cells transfected with dCas9 and TALEs targeting MUC4, relative to mock transfected cells (2 ^-ΔΔCt^) and normalised to β-actin. Primers are those described by Chen *et al.* (2013) and shown in Figure 1B. Data show means (+/- stdev) from three biological replicates. **B**) Graph showing the rise in amplified product concentration with increasing cycle number for β-actin and for MUC4. Data shown are from three technical replicates of one of the three biological replicates used in ( **A**). **C**) RT-PCR using the primers shown in Figure 1C to detect the expression of *MUC4* (top) in RNA prepared from U2OS cells and human colon mucosa. Amplification of β-actin (bottom) acts as a positive control. - reverse transcriptase (RT) and DNAse I untreated samples act as controls for the presence of genomic DNA contamination in RNA samples. **D** and **E**) UCSC Genome Browser screen shot of the genomic regions containing the *MUC20/MUC4* ( **D**) and *MUC1* ( **E**) loci and adjacent non-mucin genes. Shown below is the ENCODE H3K36me3 ChIP-seq track from U2OS cells (GEO Accession number GSM788076). Genome co-ordinates are from the hg19 assembly of the human genome.

Since it has been previously reported that targeting dCas9 to exon 2 of *MUC4* leads to partial repression of *MUC4* expression ( Chen *et al.*, 2013), we wished to assay whether *MUC4* expression is impacted similarly by the binding of TALEs or dCas9 in U2OS cells. We performed real-time (q)RT-PCR using the previously reported *MUC4* qRT-PCR primers ( Chen *et al.*, 2013). Whilst there was some modest reduction in the concentration of the MUC4 amplicons from dCas9 and TALE-transfected cells relative to mock transfected cells, this was variable between biological replicates, especially for dCas9 ( Figure 3A). However, Ct values for *MUC4* amplification were very high compared with the β-actin control ( Figure 3B) suggesting that *MUC4* expression levels may be very low in this cell line and therefore that the qRT-PCR results may be unreliable. We also noted that the previously reported ( Chen *et al.*, 2013) *MUC4* qRT-PCR primers are located entirely within exon 17 ( Figure 1B) making it hard to exclude genomic DNA contamination, or to distinguish spliced from unspliced transcripts. Indeed, we obtained the same Ct values with these *MUC4* primers in U2OS RNA samples + or -RT.

We therefore also designed RT-qPCR primers which span across the exon 3-exon 4 junction of the *MUC4* mRNA ( Figure 1C). *MUC4* encodes for a mucin, a transmembrane glycoprotein which is an important constituent of mucus. It is expressed by epithelial cells in the airway, the cervix, and the colon and is aberrantly expressed in some cancers ( Chaturvedi *et al.*, 2008). We therefore used RNA from human colonic mucosa tissue as a positive control for *MUC4* expression. RT-PCR detects a strong ~110bp *MUC4* band in the colonic mucosa sample but not in U2OS cells ( Figure 3C). There are multiple alternatively spliced isoforms of *MUC4* ( Figure 1), therefore it is possible that we were unable to detect the isoforms expressed in U2OS cells using the primers designed for RT-PCR.

Tri-methylation of histone H3K36 (H3K36me3) occurs co-transcriptionally and is enriched over the exons of expressed genes ( de Almeida *et al.*, 2011; Kim *et al.*, 2011). Analysis of ENCODE ChIP-seq data for H3K36me3 in U2OS cells shows an absence of H3K36me3 from *MUC4*, from the adjacent *MUC20* gene on chromosome 3 ( Figure 3D) and from *MUC1* located on human 1 ( Figure 3E) in contrast to the neighbouring non-mucin genes. We therefore conclude that the mucin gene family, and particularly *MUC4* is not expressed in U2OS cells.

The live cell imaging study of the *MUC4* locus ( Chen *et al.*, 2013) used the human retinal pigment epithelium (RPE) cell line, and reported that targeting dCas9 to exon 2 of *MUC4* led to partial repression of *MUC4* expression as assayed by qRT-PCR. Very significant (70–80%) repression of expression was also reported for targeting of *MUC1* with dCas9. This implies expression of the mucin genes in this cell line, which is surprising given the origin of these cells from the pigmented epithelium at the back of the eye.

**Figure 4.**
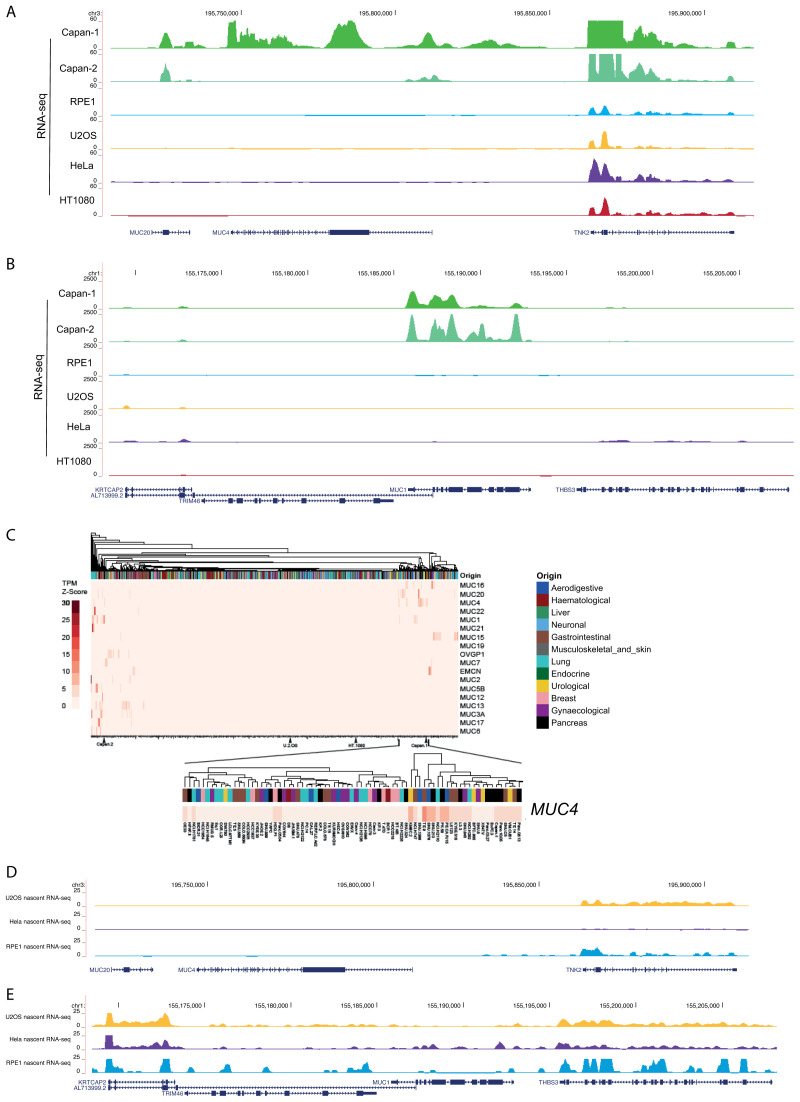
RNA-seq analysis for the mucin genes. RNA-seq data from mature mRNA at the ( **A**) *MUC4/MUC20* locus and ( **B**) *MUC1* locus for human cell lines; CAPAN-1 and CAPAN-2 pancreatic adenocarcinoma, RPE1, U2OS, HeLa and HT1080 fibrosarcomma cell lines. Data sources are detailed in Table 1. **C**) Heatmap showing z-scores for transcripts per million (TPM) for the mucin gene family obtained from RNA-seq datasets for 934 cell lines from the EBI-EMBL Expression Atlas data release 37. Cell lines were assigned an organ origin type with clustering using the default clustering method. Zoom in shows data for MUC4. **D** and **E**) Nascent (4SU) RNA-seq data at the ( **D**) *MUC4/MUC20* and ( **E**) *MUC1* loci in U2OS, HeLa and RPE cell lines. Data sources are detailed in Table 1.

To investigate this further, we searched publicly available RNA-seq datasets from RPE cells. No mature *MUC4*, *MUC20* ( Figure 4A) or *MUC1* ( Figure 4B) mRNAs were detected in these RPE datasets ( Figure 4A), making it hard to understand how meaningful the repression, reported as a consequence of dCas9 targeting at these loci, is. Consistent with previous reports ( Jonckheere *et al.*, 2004) *MUC4* expression was detected in the pancreatic adenocarcinoma cell lines CAPAN-1 and CAPAN-2.

Using publicly available datasets, we also found no evidence of mRNA expression from these mucin genes in U2OS cells or in HeLa cells ( Figure 4A and B), even though visualisation of *MUC4* transcripts using dCas13 and single molecule FISH has been recently reported in live and fixed HeLa cells, respectively ( Yang *et al.*, 2019).

Analysis of RNA-seq data for 934 cell lines, including U2OS and CAPAN cells ( Figure 4C), confirmed highly restricted expression of the entire mucin gene family, with *MUC4* expression detected in a small number of cell lines, including CAPAN-1 and 2, of gastrointestinal, urological and pancreatic origin. No expression of any mucin gene was detected in U2OS cells.

To ascertain if despite the absence of stable mucin mRNAs, there might still be transcription from the *MUC4/MUC20* loci in the cell lines examined, we assessed nascent RNA-seq (4-thiouridine/4SU-seq) data from U2OS and HeLa cells and 5-ethynyl uridine/EU-seq from RPE cells. No evidence for nascent transcription was detected from *MUC4/MUC20* in data from any of these cell lines ( Figure 4D).

## Discussion

The ability to detect endogenous gene loci in mammalian cells is an important goal and, the ability to study these genes during the act of transcription is key to understanding both the chromatin dynamics associated with transcription and the spatial organisation of these genes relative to the components of the transcriptional machinery. Whilst many groups are exploring ways to improve the signal:noise problems inherent in visualising a single-copy gene, the mammalian *MUC4* gene could be an excellent model since the repetitive nature of the sequences in exon 2 and intron 3 maximises the detection of fluorescent signal from molecules targeted to this locus – e.g. through dCas9 or TALEs ( Chen *et al.*, 2013) ( Figure 1). A similar approach may be feasible for other genes, such as *FLG* (encoding filaggrin), which also contain tandem repeats ( Brown *et al.*, 2012).

There are several studies reporting repression of the *MUC4* (and *MUC1*) genes upon binding by dCas9 ( Chen *et al.*, 2013) and visualisation of *MUC4* transcripts using dCas13 ( Yang *et al.*, 2019). However, our data reported here suggest a re-examination of the conclusions from these reports is required in order to understand, for example, whether these discrepancies arise from breakthrough transcription in a very small fraction of cells, from differences between batches of cell lines, or from differences in the ability to detect transcription using different methods. Our data also suggest that other cell lines that robustly express mucin genes might be a better system to employ for live cell imaging studies. 

## Data availability

### Underlying data

NCBI Gene Expression Omnibus: Sources of H3K4me3. Accession number GSM871043; https://identifiers.org/geo:GSM871043.

NCBI Gene Expression Omnibus: Sources of H3K36me3. Accession number GSM788076; https://identifiers.org/geo:GSM788076.

NCBI Gene Expression Omnibus: Sources of mRNA RNA-seq data for Capan-1 and 2 cells. Accession number GSE79669; https://identifiers.org/geo:GSE79669.

NCBI Gene Expression Omnibus: Sources of mRNA RNA-seq data for U2OS. Accession number GSE162163; https://identifiers.org/geo:GSE162163.

NCBI Gene Expression Omnibus: Sources of mRNA RNA-seq data for RPE1. Accession number GSE98541; https://identifiers.org/geo:GSE98541.

NCBI Gene Expression Omnibus: Sources of mRNA RNA-seq data for HeLa. Accession number GSE90235; https://identifiers.org/geo:GSE90235.

NCBI Gene Expression Omnibus: Sources of mRNA RNA-seq data for HT1080. Accession number GSE78653; https://identifiers.org/geo:GSE78653.

NCBI Gene Expression Omnibus: Sources of nascent RNA-seq data for U-2OS. Accession number GSE162264; https://identifiers.org/geo:GSE162264.

NCBI Gene Expression Omnibus: Sources of nascent RNA-seq data for RPE1. Accession number GSE137448; https://identifiers.org/geo:GSE137448.

NCBI Gene Expression Omnibus: Sources of nascent RNA-seq data for HeLa. Accession number GSE128753; https://identifiers.org/geo:GSE128753.
