# Supplementary material for: MUC4 is not expressed in cell lines used for live cell imaging
Source: Wellcome Open Res. 2021 Nov 19;6:265. Originally published 2021 Oct 13. [Version 2] doi: 10.12688/wellcomeopenres.17229.2 (PMC8567686; doi:10.12688/wellcomeopenres.17229.2)
Supplement: Peer review history

# Peer review: Reviewer response for version 1

**DOI:** 10.21956/wellcomeopenres.19041.r46425

**Published:** 2021-11-03

**Authors:** Jonathan Chubb

**Affiliations:** 1 MRC Laboratory for Molecular Cell Biology and Department of Cell and Developmental Biology, University College London, London, UK

This is a very interesting contribution to the technology development of Cas9/Talen tools for imaging and controlling gene expression and chromatin dynamics. A variety of approaches have been used over the past 35 years, starting with the imaging of DAPI and EtBr stained nuclei, through to the integration of repeats of bacterial repeats and their detection using repeat binding proteins. These methods have limits in resolution and potential disruption of the underlying template. The potential to detect single loci and their products without disrupting the underlying locus promises more precise measurements of a more physiological chromatin state. Many initial studies using Talens and Cas9 tools have focussed on the MUC4 gene, as its repetitive nature allows the accumulation of more fluorescence at the site of transcription. This new study indicates that MUC4 is not expressed in most of the cell lines under study, which limits the interpretation of any chromatin dynamics inferred, and the magnitude of any unwanted effects on the endogenous locus inferred using Cas9 and Talens to image chromatin.

 The manuscript is generally well written, and the conclusions unequivocal. The statistical analysis is limited, but the data are so clear, this probably warrants little consideration. The text in the legend for Figure 4E is missing and should be added. Perhaps also some discussion of an alternative target for imaging, such as an abundantly expressed gene with lots of repeats, such as Filaggrin.

Is the work clearly and accurately presented and does it cite the current literature?

Yes

If applicable, is the statistical analysis and its interpretation appropriate?

Yes

Are all the source data underlying the results available to ensure full reproducibility?

Partly

Is the study design appropriate and is the work technically sound?

Yes

Are the conclusions drawn adequately supported by the results?

Yes

Are sufficient details of methods and analysis provided to allow replication by others?

Yes

Reviewer Expertise:

Single cell gene expression, imaging, development.

I confirm that I have read this submission and believe that I have an appropriate level of expertise to confirm that it is of an acceptable scientific standard.

# Response

**Published:** 2021-11-17

**Authors:** Wendy Bickmore

**Affiliations:** MRC Human Genetics Unit, UK

We thank the reviewer for pointing out the omission in the legend to Figure 4E, this has now been corrected in a revised version. We also thank the reviewer for pointing out other genes with internal tandem repeats, and we have now added a statement about alternative gene targets such as Filaggrin .

# Peer review: Reviewer response for version 1

**DOI:** 10.21956/wellcomeopenres.19041.r46423

**Published:** 2021-10-22

**Authors:** Thomas Sexton

**Affiliations:** 1 CNRS UMR7104, INSERM U1258, IGBMC, Université de Strasbourg, Strasbourg, France

This clearly written report convincingly shows lack of MUC4 expression in the cell lines used in previous live imaging studies, and the authors rightly highlight the care needed in developing and validating such studies in the future.

 It would be nice if the authors could be a bit more explicit in their take on what this means for the potential impact of dCas9 and/or TALEs on expression of the genes they are being used to image. Are there other cases unrelated to MUC4 in the literature, showing transcription perturbation by these tools, and are they more believable? Or do the authors believe that the impact of the tools on transcription has not yet been adequately tested?

Is the work clearly and accurately presented and does it cite the current literature?

Yes

If applicable, is the statistical analysis and its interpretation appropriate?

Not applicable

Are all the source data underlying the results available to ensure full reproducibility?

Yes

Is the study design appropriate and is the work technically sound?

Yes

Are the conclusions drawn adequately supported by the results?

Yes

Are sufficient details of methods and analysis provided to allow replication by others?

Yes

Reviewer Expertise:

Molecular biology; nuclear organisation

I confirm that I have read this submission and believe that I have an appropriate level of expertise to confirm that it is of an acceptable scientific standard.

# Response

**Published:** 2021-11-16

**Authors:** Wendy Bickmore

**Affiliations:** MRC Human Genetics Unit, UK

We thank this reviewer for these comments. We do indeed think that the impact of dCas9 or TAL effector tools on DNA templated transactions, including transcription, have not been systematically tested to date.
